# Using telemedicine to manage a patient with a hypertensive emergency due to pheochromocytoma

**DOI:** 10.1530/EDM-23-0033

**Published:** 2023-07-04

**Authors:** Seigo Konishi, Mitsuhiro Kometani, Ko Aiga, Daisuke Aono, Takahiro Nohara, Hiroko Ikeda, Shigehiro Karashima, Yoshiyu Takeda, Takashi Yoneda

**Affiliations:** 1 Department of Health Promotion and Medicine of the Future, Graduate School of Medical Sciences, Kanazawa University, Kanazawa, Ishikawa, Japan; 2 Integrative Cancer Therapy and Urology, Division of Cancer Medicine, Graduate School of Medical Science, Kanazawa University, Kanazawa, Ishikawa, Japan; 3 Department of Diagnostic Pathology, Kanazawa University Hospital, Kanazawa, Ishikawa, Japan; 4 Endocrine and Diabetes Center, Asanogawa General Hospital, Kanazawa, Japan

**Keywords:** Adolescent/young adult, Female, Asian - Japanese, Japan, Adrenal, Endocrine-related cancer, Novel treatment, July, 2023

## Abstract

**Summary:**

The COVID-19 pandemic has led to the emergence of telemedicine on a global scale. In endocrinology, telemedicine has mainly been used in relation to chronic diseases, including diabetes. Herein, we report the case of an 18-year-old woman with a hypertensive emergency due to a pheochromocytoma who was quickly diagnosed and treated using telemedicine. The patient was referred to a cardiovascular hospital because of fatigue and sweating that did not improve with carvedilol. She had fluctuating blood pressure and tachycardia. Subsequently, since her thyroid function was normal, endocrine hypertension not due to thyroid dysfunction was suspected; a case consultation was made by phone to our clinic. Plain computed tomography (CT) was recommended owing to the high possibility of a pheochromocytoma; the CT scan showed an adrenal tumor with a 30 mm diameter. To assess her condition, endocrinologists, together with the attending doctor, interviewed her and her family directly using an online tool to obtain detailed information. We thus determined that she was at risk of a pheochromocytoma crisis. She was transferred to our hospital immediately for treatment, was diagnosed with pheochromocytoma, and underwent surgery. Telemedicine, especially involving doctor-to-patient with doctor consultations, can be effective in treating rare and emergent medical conditions such as pheochromocytoma crisis.

**Learning points:**

- Telemedicine can be used for chronic diseases and emergency conditions.
- Online doctor-to-patient with doctor (D-to-P with D) consultations are useful when the expert opinion of a highly specialized physician present in a different geographical location is required.
- Telemedicine, especially D-to-P with D online consultations, can be effectively used for the diagnosis of rare and emergent medical conditions, such as pheochromocytoma crisis.

## Background

Pheochromocytoma is a rare cause of hypertensive disease. The prevalence of pheochromocytoma crisis in patients with pheochromocytoma is 3%, and the mortality rate among patients with pheochromocytoma crisis is approximately 17% (1). Endocrinologists are easily able to diagnose pheochromocytoma by thorough history taking, physical examination, and biochemical and imaging studies; however, non-endocrinologists can face challenges in diagnosing the disease.

During the COVID-19 pandemic, the use of telecommunication and electronic information technologies, including telemedicine, gained popularity (2). Telemedicine provides patients, their families, and medical practitioners safe access to a variety of treatment options, including primary care consultations, nutrition therapy, and physical therapy.

Telemedicine is usually performed between doctors and patients (doctor to patient: D-to-P). The subtypes of telemedicine with a healthcare provider include doctor-to-patient with nurse and doctor-to-patient with doctor (D-to-P with D).

In endocrinology, telemedicine has mainly been used in relation to chronic diseases, such as diabetes. Regarding previously managed cases of pheochromocytoma via telemedicine, one study reported on preoperative alpha blocker dosage managed using e-mail telemedicine to reduce both the number of preoperative visits and the mean time from treatment initiation to surgery (3). Another case study reported long-term (7–18 weeks) medical management via videoconference and telephone during the stable period prior to adrenalectomy at the peak of COVID-19 prevalence (4). Both reports described patient management when the patient was in a stable condition.

Herein, we report the case of a young woman with hypertensive emergency due to a pheochromocytoma; she was rapidly diagnosed and treated through a D-to-P with D online consultation.

## Case presentation

An 18-year-old woman presented to her family physician with complaints of fatigue and sweating. She had no relevant medical history, but her mother had a history of chronic thyroiditis. From the age of 15 years, she was aware of intermittent palpitations and headaches, without any specific triggers. At the time of her visit, her systolic blood pressure was unstable, ranging from 140 to 200 mmHg. Carvedilol (10 mg/day) was administered, but her symptoms did not improve. Therefore, she was referred to a cardiology hospital where she presented with a fluctuating systolic blood pressure between 160 and 250 mmHg, persistent hyperhidrosis, and sinus tachycardia (130 beats/min).

Secondary hypertension due to an endocrine disease was suspected based on her age and high blood pressure. However, there was no endocrinologist at the hospital, the attending doctor made a phone call to our institution and provided information about the patient’s condition, excluding personal information. Based on the symptoms described, pheochromocytoma and thyroid disease were listed as differential diagnoses; however, her laboratory data showed no thyroid dysfunction. It takes several days to obtain catecholamine levels and an immediate diagnosis was needed, and carvedilol administration might have worsened the symptoms of pheochromocytoma; therefore, we advised the doctor to perform whole-body computed tomography (CT) for tumor detection.

## Investigation

**Figure 1:**
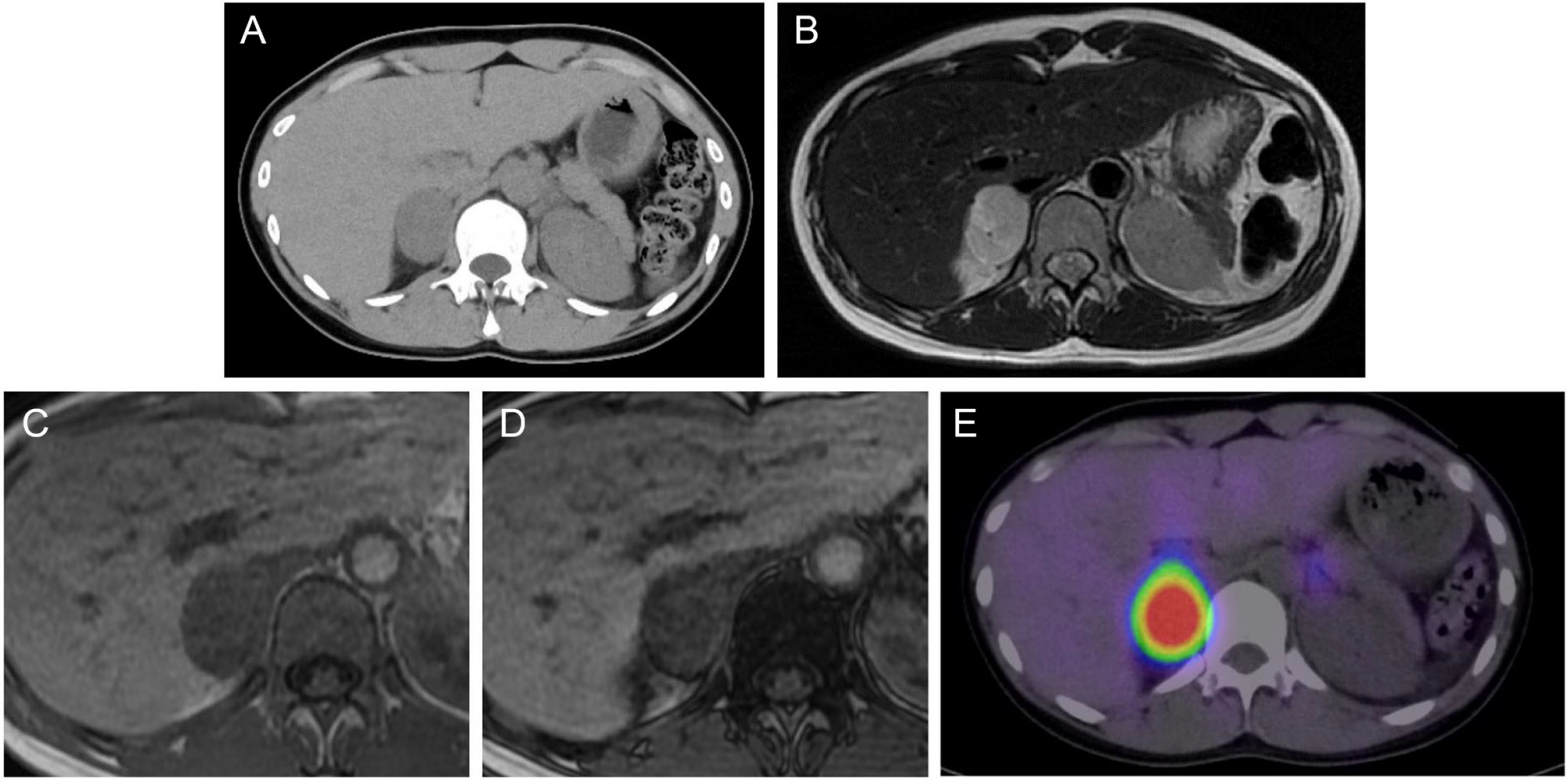
Images of the right adrenal tumor. (A) Right adrenal tumor measuring 38 × 26 mm (computed tomography number: 40 HU). (B–D) Right adrenal tumor showing high signal on T2-weighted images and no signal loss on T1-weighted images from the in-phase to the out-of-phase. (B) T2-weighted images. (C) T1-weighted images (in-phase). (D) T1-weighted images (out-of-phase). (E) Iodine-123 metaiodobenzylguanidine scintigraphy showing high uptake, consistent with the finding of a tumor.

**Figure 2:**
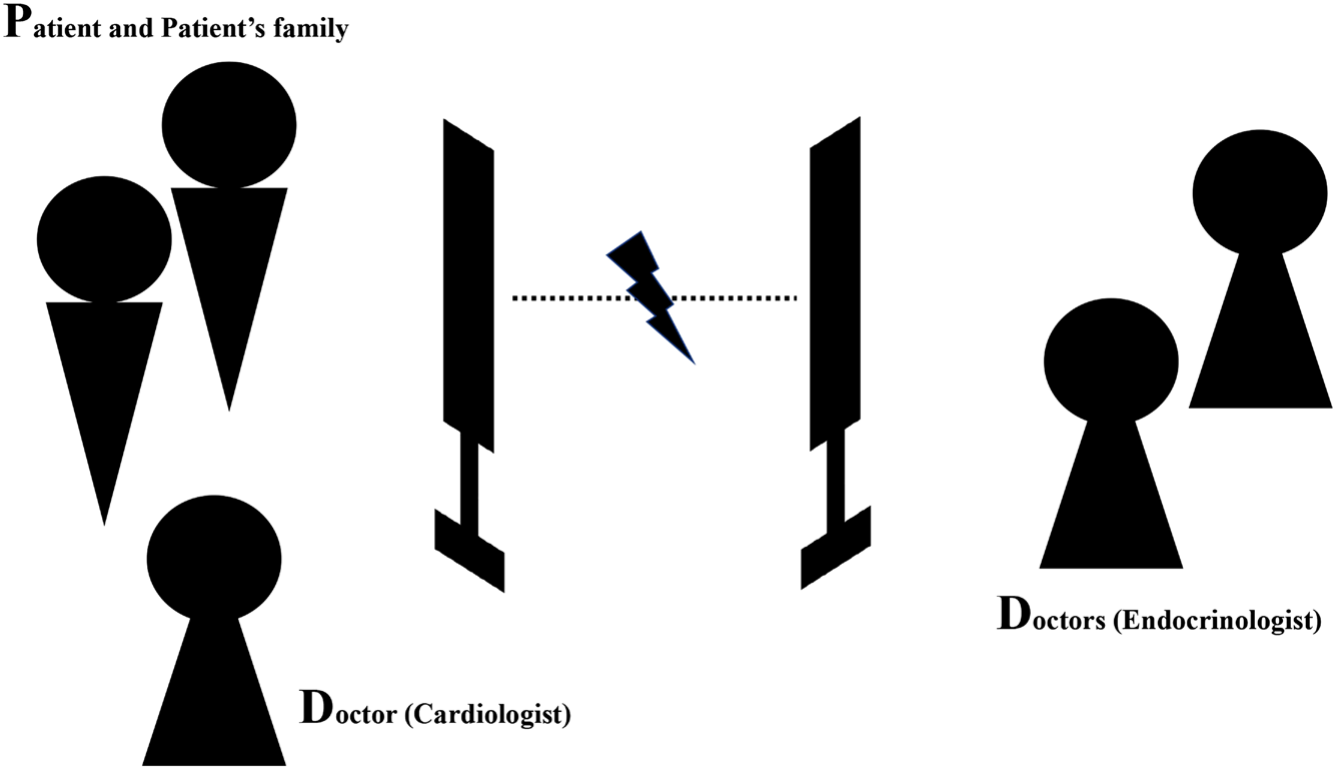
Telemedicine image in this case (D-to-P with D). D-to-P with D, doctor-to-patient with doctor.

**Figure 3:**
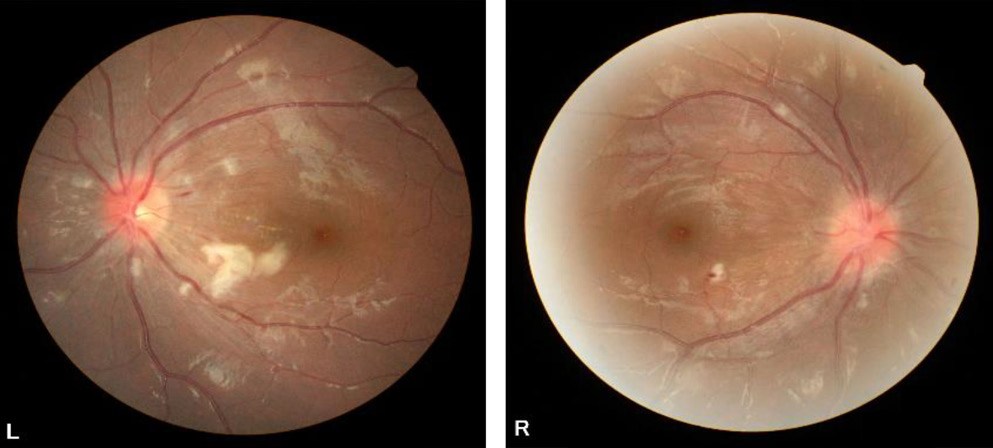
Photographic images of the fundus.

CT scan revealed a tumor measuring 38 × 26 mm in diameter on the right adrenal gland, indicating the presence of a pheochromocytoma (Fig. 1). Transfer to an institution with a specialist such as ours was considered, but the patient was deemed to be in an unstable condition. Therefore, because of the patient's young age and for the purpose of assessing the severity of the disease and explaining the future course of action to the patient and her family, we used an online system that allowed us, along with the attending doctor, to examine the patient directly via a screen. Telemedicine was conducted using a real-time video call, with the patient, her family, and a cardiologist participating on the patient’s side and several endocrinologists participating on the endocrinologist’s side (Fig. 2). We obtained detailed information by interviewing the patient and her family. We ascertained her state of consciousness, assessed her disease severity, and visually confirmed her complaint of excessive sweating. The patient and her family were informed that she was at risk of developing pheochromocytoma crisis, and prompt treatment by specialists was advised. Hence, she was transferred to our hospital 3 h after having been admitted to the cardiology hospital. Since the patient and her family had been informed of her condition as well as the details of her examination and treatment during the online consultation, we initiated treatment in the emergency department immediately. Physical examination revealed no neurofibromas or café-au-lait macules. She weighed 49 kg, with a body mass index of 20.1 kg/m^2^. Her blood pressure in the sitting position was 117/93 mmHg, and her pulse rate was 128/min. In the supine position, her blood pressure was 189/133 mmHg, and her pulse rate was 126/min. She was conscious and her body temperature was 36.8°C. Bowel sounds were reduced. Her skin was moist with visible sweating. Blood tests revealed hemoconcentration (hemoglobin: 15.5 g/dL; hematocrit: 44.3%) and hypokalemia (K: 3.2 mEq/L). Urinalysis results were negative for urinary proteins. A 24-h urine test result revealed high urinary noradrenaline (4590 μg/day, reference range (RR): 29–120 μg/day) and urinary normetanephrine (6.70 mg/day, RR: 0.10–0.28 mg/day) levels in a 24-h urine collection sample. Her electrocardiogram showed sinus rhythm with a heart rate of 108/min, left axis deviation and left ventricular hyper-potentiation, and a negative T-wave in V2–6, with chest induction. Echocardiography revealed overall wall thickening and mild wall motion loss, but there were no findings suggestive of significant valvular disease, pericardial effusion, pleural effusion, or takotsubo cardiomyopathy. Chest radiography revealed no special features. Fundus photographs revealed H4S0 (congested papillae and no sclerotic changes) in both eyes, with bilateral optic nerve papillae, soft leukoderma, and mottled and linear hemorrhages (Fig. 3). Abdominal magnetic resonance imaging showed a well-defined 35-mm diameter tumor on the right adrenal gland with a high signal on T2-weighted images and no loss of signal intensity between the in-phase and out-of-phase on T1-weighted images. Consistent accumulation was observed in the right adrenal tumor on iodine-123 metaiodobenzylguanidine scintigraphy.

## Treatment

The patient was diagnosed with a hypertensive emergency due to pheochromocytoma. Treatment was initiated with continuous phentolamine infusion (2 mg/h), oral doxazosin (4 mg/day), and saline infusion (2000 mL/day), which improved her palpitation and sweating symptoms. On day 2, propranolol 30 mg/day was started, doxazosin was titrated, and phentolamine and external fluids were tapered off. On day 6, phentolamine was discontinued. Blood pressure of 130–160/70–80 mmHg, sitting heart rate of 60–70/min, and standing heart rate of 70–80/min were achieved with doxazosin (16 mg/day) and propranolol (60 mg/day). Adrenalectomy was performed on day 25. The right adrenal gland weighed 25 g; measured 6 cm; and contained a well-defined, golden, solid mass measuring approximately 4 cm. On histological examination, the tumor showed large cell nests with intermediate cellularity of the noradrenergic type and was classified as a moderately differentiated tumor with a score of 3, based on the grading system for adrenal pheochromocytoma and paraganglioma. Succinate dehydrogenase subunit B (SDHB) immunostaining revealed tumor areas with attenuated or absent staining.

## Outcome and follow-up

As our patient presented with a relatively young-onset pheochromocytoma and the immunostaining results suggested the presence of SDHB mutation, the risk of metastasis was considered to be high. At the 2-year follow-up, no recurrence was reported.

## Discussion

The acute phase of the endocrine disease in our patient was successfully managed using telemedicine. Telemedicine may be useful in emergency conditions. Cost savings, improved quality of care, low patient transfer rates, and low mortality rates are some of the benefits of implementing telemedicine in emergency departments; however, technical, legal, and ethical/policy issues have been noted (5).

Compared with conventional referral consultations through documents or telephone calls, a D-to-P with D online consultation has many advantages (Table 1). First, at a D-to-P with D online consultation, the patient is also present. This may increase the patient's satisfaction and reduce the burden of explanation on the family physician, which may be beneficial in rare emergency cases that require highly specialized care, as in our case reported here. Direct contact with a specialist helps patients and their families understand the need for in-depth testing and treatment as an inpatient or during transfer between centers. Moreover, direct online monitoring of patients and sharing test results with referring physicians allow specialists to determine the severity of a condition and early post-transfer testing and treatment plans.

**Table 1:** Comparison of consultations when no relevant specialist is available at a hospital.

	Documents via postal service	Documents via fax or e-mail	Telephone	Telemedicine
Days required	A few days	Almost immediate or a few days	Immediate	Immediate; however, pre-setting is required
Real time or differential time	Differential time	Differential time	Real time	Real time
Equipment penetration	Almost all hospitals	Almost all hospitals	Almost all hospitals	Both hospitals must have facilities and internet access for telemedicine
Target	Doctor-to-doctor	Doctor-to-doctor	Doctor-to-doctor	Doctor-to-patient with doctor
Availability of visual information on the patient	No	No	No	Yes
Necessity of obtaining consent	No	No	No	Yes
Reflection in the medical record	Easy	Easy	Separate medical record entry required	Separate medical record entry required*

*Legislation required.

Despite the benefits of telemedicine, some existing challenges may reduce this method’s acceptability. One limitation is the need for two healthcare professionals to be present for online consultations. Both doctors involved need to be proficient in setting up the necessary equipment. The speed and stability of the communication lines greatly affect the quality of these online consultations. Privacy issues such as the possibility of the communication being intercepted by third parties or the consultation being recorded with or without permission should also be considered. Finally, the possible legal liabilities associated with online treatment have not yet been clarified.

Currently, academic institutions and various countries are beginning to establish laws and regulations for telemedicine to ensure that patients are not disadvantaged. In Japan, in cases such as ours, where the general practitioner refers a patient to a medical institution providing specialized medical care (including cases where necessary collaboration is in place and D-to-P with D is used), online medical care can be applied from the initial consultation (https://www.mhlw.go.jp/content/000889114.pdf, (In Japanese). Published March 2018, partially revised March 2023,Accessed April 26, 2023). D-to-P with D is suitable for situations when a diagnosis or surgery is required by a highly skilled physician present in a different location. In the United States, the situation varies from state to state, including whether telemedicine is covered for both Medicaid and private payers or for Medicaid only, whether a state license is required to practice telemedicine, and whether voice-only telemedicine services, excluding mental health services, are allowed once a public health emergency has ended. The situation also differs among countries (https://connectwithcare.org/wp-content/uploads/2022/08/The-Roadmap-to-Telehealth-Efficacy-Care-Health-and-Digital-Equities.pdf. Published July 2022, Accessed April 26, 2023). In most European countries and in Israel, telemedicine is permitted (as of July 2021), with most countries having no explicit laws regulating telemedicine (https://biolegis.com/wp-content/uploads/2021/09/EU-Legal-Framework-on-Telemedicine.pdf. Published July 2021, Accessed April 26, 2023). In Israel, under the State Health Insurance Law, each resident is entitled to basic healthcare, including free access to primary care clinics and the use of telemedicine (6).

Appropriate regulations for D-to-P with D consultations need to be developed and promoted by governments and academic institutions. However, as noted in a previous report, ‘using a combination of in-person and telehealth care’ and ‘developing strong relationships and norms for bidirectional communication with referring clinicians’ are important aspects (7). Telemedicine, including D-to-P with D consultations, should first be performed at facilities where it is feasible, and it is important to accumulate evidence pertaining to this practice. In summary, our case report shows that a young woman with a hypertensive crisis due to a pheochromocytoma benefited from an online D-to-P with D consultation. Online D-to-P with D consultations might be useful when highly specialized physicians need to make diagnostic and policy decisions on urgent and low-frequency conditions from a different location.

## Declaration of interest

The authors declare that there is no conflict of interest that could be perceived as prejudicing the impartiality of the research reported.

## Funding

This study did not receive any specific grant from any funding agency in the public, commercial, or not-for-profit sector.

## Patient consent

Written informed consent for publication of the clinical details and images was obtained from the patient.

## Author contribution statement

SK, MK, and KA wrote the paper. MK, KA, and DA made the figures and tables. TN was involved in surgery and HI in pathology. SK, YT, and TY supervised the case report. All authors reviewed the manuscript.
